# Implementation and clinical evaluation of an in-house thoracic auto-segmentation model for 0.35 T magnetic resonance imaging guided radiotherapy

**DOI:** 10.1016/j.phro.2025.100819

**Published:** 2025-08-05

**Authors:** Nikolaos Delopoulos, Sebastian Marschner, Elia Lombardo, Marvin F. Ribeiro, Paul Rogowski, Christoph Losert, Tobias Winderl, Shadi Albarqouni, Claus Belka, Stefanie Corradini, Christopher Kurz, Guillaume Landry

**Affiliations:** a Department of Radiation Oncology, LMU University Hospital, LMU Munich, Munich, 81377, Germany; b German Cancer Consortium (DKTK), partner site Munich, a partnership between DKFZ and LMU University Hospital Munich, Munich, 81377, Germany; c Bavarian Cancer Research Center (BZKF), Munich, 81377, Germany; d Clinic for Diagnostic and Interventional Radiology, University Hospital Bonn, Bonn, 53127, Germany; e Helmholtz AI, Helmholtz Munich, Neuherberg, 85764, Germany; f Department of Radiation Oncology (Maastro), GROW School for Oncology and Reproduction, Maastricht University Medical Centre, Maastricht, 6229, The Netherlands; g Department of Radiology and Nuclear Medicine, Mental Health and Neuroscience (MHeNs), Faculty of Health Medicine and Life Sciences, Maastricht University Medical Center, Maastricht, 6229, The Netherlands

**Keywords:** MRI, Radiation therapy, MR-linac, Segmentation, Auto-segmentation, Deep learning, Artificial intelligence, Organs-at-risk, Magnetic resonance imaging-guided radiotherapy, Radiotherapy planning, Medical image analysis, Contour correction, Workflow efficiency, Clinical integration, Automation in radiotherapy, Lung cancer, Medical AI deployment

## Abstract

**Background and Purpose:**

Magnetic resonance imaging-guided radiotherapy (MRgRT) facilitates high accuracy, small margins treatments at the cost of time-consuming and labor-intensive manual delineation of organs-at-risk (OARs). Auto-segmentation models show promise in streamlining this workflow. This study investigates the clinical applicability of a set of thoracic OAR segmentation models for baseline treatment planning in lung tumor patients. We investigate the use of the models for treatment at a 0.35 T MR-linac, assess their potential to reduce physician workload in terms of time savings and quantify the extent of required manual corrections, providing insights into the value of their integration into clinical practice.

**Materials and Methods:**

Deep-learning based auto-segmentation models for 9 thoracic OARs were integrated into the MRgRT workflow. Two groups of 11 lung cancer cases each were prospectively considered. For Group 1 auto-segmentation contours were corrected by physicians, for Group 2 manual contouring according to standard clinical workflows was performed. Contouring times were recorded for both. Time savings between the groups as well as correlations of the extent of corrections to correction times for Group 1 patients were analyzed.

**Results:**

The model performed consistently well across all Group 1 cases. Median contouring times were reduced for six out of nine OARs leading to a reduction of 50.3 % or 12.6 min in median total contouring time.

**Conclusion:**

Feasibility of auto-segmentation for baseline treatment planning at the 0.35 T MR-linac was shown with significant time savings demonstrated. Time saving potential could not be estimated from model geometric performance metrics.

## Highlights

• Clinical evaluation of artificial intelligence based auto-contouring.
• Application on 0.35 T Magnetic Resonance guided Radiation Therapy treatment planning.
• Comparative evaluation of potential time savings with use of artificial intelligence.
• In-house development and clinical implementation of applied models.
• Significant savings in total contouring time.

## 1 Introduction

Magnetic resonance linear accelerators (MR-linacs) and magnetic resonance imaging-guided radiotherapy (MRgRT) brought a major technological advancement in cancer treatment [1]. Compared to cone-beam computed tomography (CBCT)-guided stereotactic body radiation therapy (SBRT), MRgRT offers superior soft-tissue contrast and eliminates imaging radiation dose [2], [3]. This enables precise tumor targeting and sparing of healthy tissues [4].

During planning, computed tomography (CT) and magnetic resonance (MR) scans are acquired and deformably registered. Physicians then manually contour target volumes and organs-at-risk (OARs) on MR images (MRI). The CT scans are used to derive the electron density needed for optimizing the radiation treatment plan [5], [6].

Manual delineation of target volumes and OARs within this workflow is a repetitive, time-consuming and labor-intensive task. It is also prone to variability between and within observers, which can affect the consistency and quality of treatment. This variability poses a significant challenge, as precision and reproducibility in contouring are essential for effective radiotherapy [7].

These limitations have spurred the development of auto-segmentation (AS) models, which aim to streamline the contouring process while maintaining or improving accuracy [8], [9]. AS models leverage deep learning (DL) techniques to generate contours with accuracy comparable to those created manually by physicians and have runtimes ranging from minutes to seconds [10]. This time is negligible compared to the duration of MRgRT planning, particularly since it occurs offline and does not consume physician time. By automating the most time-consuming step of the workflow, such models shift the physician’s role from manual delineation to the verification and correction of the automatically generated contours [11]. While there are several MRgRT AS models for the pelvic [12], [13], abdominal [14] and head and neck regions [15], only a few models have been reported for the thoracic region [16], [17]. Ribeiro et al. reported Dice similarity coefficients (DSC) ranging from 0.78 to 0.96 depending on the OAR when using a 3D UNet [16]. In an offline review setting, a clinician deemed that only 19 thoracic OAR segmentations out of 129 required editing prior to clinical use [16]. Chekroun et al. reported similar performance when using their Efficient-UNet [17].

While geometric accuracy is an important factor [18], it is at least equally important to evaluate models on their potential to enhance clinical efficiency. Their impact on reducing the time spent on contouring tasks should play a crucial role in decision-making regarding their clinical integration [19], [20]. This is infrequently addressed in AS literature, particularly within the clinical context, where physician awareness of the direct use of contours for treatment imposes more stringent quality standards compared to offline evaluations of contour accuracy. It may also be possible that offline reviews as reported in Ribeiro et al. [16] may lead to different results than during real-world clinical evaluation, as observed in the case of automatic treatment planning [21].

This contribution aims to assess the clinical value of DL-based AS models specifically applied to the MRgRT planning workflow for lung cancer patients treated with a 0.35 T MR-linac. The models, trained on data from the same system and institution, were implemented in the clinical setting. Performance was evaluated by comparing the time and accuracy of contour generation between AS-assisted workflows and manual ones. This comparison will help clarify the extent to which such models can reduce the manual burden on clinicians and improve workflow efficiency, while ensuring that the accuracy and quality of contours meet the standards required for patient care.

By focusing on both geometric performance and time savings, this study provides insights into the practical applicability of AS models in MRgRT.

## 2 Material and methods

### 2.1 Thoracic segmentation models

A set of 6 thoracic single-organ AS models was implemented without re-training in the clinical setting [16]. The models were implemented using PyTorch [22] and MONAI [23]. 122 MRIs of lung cancer patients were used – 80, 19 and 23 for training, validation and testing, respectively – all manually delineated by physicians of the LMU Department of Radiation Oncology. The original publication reported geometric performance analysis using the DSC and the Hausdorff distance (HD), with median DSC of 0.96, 0.96, 0.94, 0.90, 0.88 and 0.78 and median of the 95th percentile HD (HD95) of 3.9, 5.3, 5.8, 3.0, 2.6 and 3.5 mm for left lung, right lung, heart, aorta, spinal canal and esophagus, respectively. The model’s clinical usability was further retrospectively assessed by a physician, who found that out of 129 AS contours, 85 required no correction, 25 were immediately usable for treatment planning, 15 required minor and 4 major corrections.

In this study, we added 3 single-organ models for the left bronchial tree, right bronchial tree and trachea, completing a clinically usable set of OARs for lung cancer patients. Using the same architecture, training cohort, and hyperparameters as the original models, they were trained on 37/13/13, 39/8/7, and 39/12/13 train/validation/test cases, respectively. Evaluated on each test set, they showed median DSCs of 0.66, 0.58 and 0.59 and median HD95 of 7.7, 6.0 and 5.2 mm respectively.

All implementation details of the models were kept identical to the original study, with an added automation layer to facilitate data transfers, queuing and automatic model execution.

### 2.2 In-house segmentation service

**Fig. 1:**
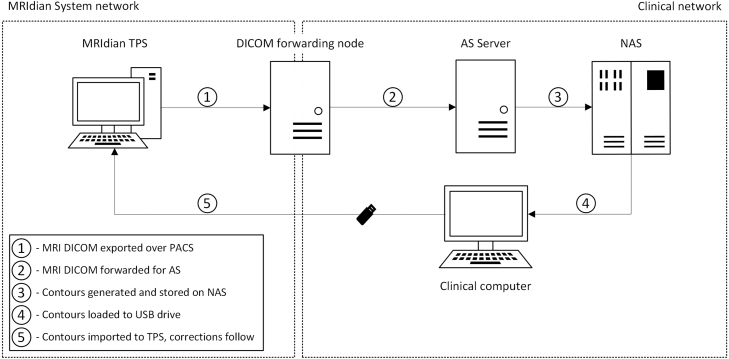
Computer network architecture diagram illustrating components and operations as described in Section 2.2. Dotted boxes represent firewall-separated networks, arrows the flow of data between network components.

A semi-automated pipeline was developed, linking the MRIdian treatment planning system (TPS, version 5.3.6.11) to a segmentation server (See Fig. 1). We prioritized minimizing the additional workload for clinicians while maintaining the integrity of existing clinical machine configurations. This pipeline spans two computer networks: the local MR-linac system network, which manages intra-system device communication, and the broader internal clinical network. These networks are connected exclusively through a DICOM node, enabling the forwarding of DICOM series from the MRIdian TPS to DICOM nodes in the clinical network. Note that the current version of the MRIdian TPS only supports contour import from local files.

To address security requirements, a docker-based [24] architecture was developed, consisting of three independent containers: a DICOM node for receiving MRI series, an execution environment for PyTorch-based AI models, and a data recorder which stores model results on a network-attached storage (NAS) server. The architecture was deployed on a server running an immutable Linux distribution with strict firewall rules, which ensure that any communications aside from incoming DICOM series and write operations to the NAS, are blocked.

The resulting automated segmentation workflow proceeds as follows:

1. The MRI planning scan is exported from the MRIdian TPS to the intermediate node via DICOM export functionality.
2. The scan is forwarded to the AS server via the intermediate node’s GUI.
3. On the AS server, a sequence of operations is triggered automatically:
  (a) Each of the nine sub-models is executed sequentially.
  (b) The resulting contours are merged into an RTSTRUCT DICOM file.
  (c) The file is saved onto a network drive accessible to all computers connected to the clinical network.
4. The file is manually transferred from a clinical computer to a USB drive and subsequently moved to the MRIdian TPS.
5. The contours are imported using the “import from file” functionality and can then be adjusted by a physician in the MRIdian TPS.

The manual operations in step 4 are unavoidable given the current capabilities of the components involved and the limitation of not modifying systems in clinical use. Nonetheless, the semi-automated nature of the pipeline, combined with the docker-based architecture and robust security measures, considerably enhances the efficiency of the workflow.

### 2.3 Data collection

A prospective cohort of 22 lung cancer cases not included in AS model development and testing, treated between March 2023 and May 2024 with images acquired using the 3D balanced steady state free-precession sequence and a $1.5\times 1.5\times 3\;{\mathrm{mm}}^{3}$ voxel size was divided into two groups to evaluate the impact of AS in the treatment planning workflow. Two senior physicians were involved in the study, with patients of both groups split among them based on their workload and availability on a day-to-day basis. Only one physician was involved with each patient at the contouring stage of treatment planning. Group 1 (AS) consisted of 11 cases. For this group, physicians submitted planning MRIs to the in-house AS service, which generated contours for the nine OARs. Upon their import to the TPS, the time required by the physician per OAR to verify or correct the model output for clinical application was recorded using a stopwatch. Physicians were instructed to measure time spent on editing the contours, pausing the timing when other tasks or distractions were involved. Time spent using the necessary interface and execution times of the models (totaling approximately 3 min) was not taken into account, simulating a system where these would happen in the background after each image acquisition. In contrast, for Group 2, comprising 11 cases, only time tracking was introduced with the physician otherwise performing the standard clinical manual segmentation routine. Again, the times for all individual OARs were recorded. For both groups, the standard treatment planning workflow was resumed after the timing stage. All patient data used in this study was collected in a prospective observational study (ethics committee study number 20–291). For Group 1, the degree of correction required for the AS contours was evaluated by calculating several metrics [25], [26]: the DSC, surface DSC with a 3 mm threshold, HD, average HD (avgHD), HD95 and total added path length with a 3 mm threshold (totalAPL) between the model-generated and the corrected contours used for treatment. All metric calculations were implemented under the plastimatch and platipy frameworks [27], [28]. For Group 1, the relationship between these metrics and the times necessary for contour corrections was also investigated by calculating the Spearman’s rank correlation coefficient between the various scores and correction times and corresponding two-tailed significance values with a p<0.05 threshold. Finally, a one-tailed Mann–Whitney U test with a significance threshold of p<0.025 was performed on the per-organ and total correction versus manual segmentation times to determine the potential statistical significance of the time savings.

## 3 Results

### 3.1 Time efficiency in contouring

**Fig. 2:**
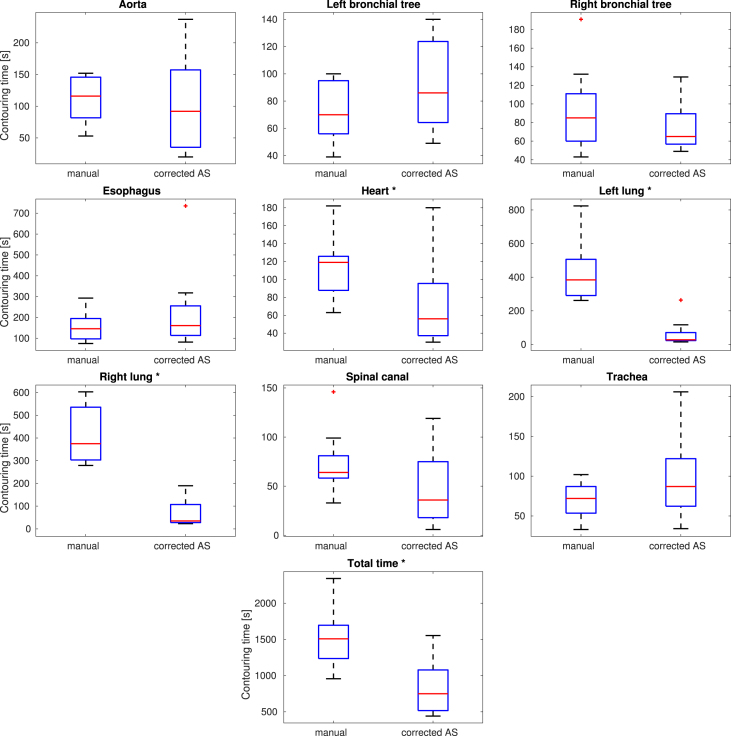
Per organ manual segmentation versus correction times in seconds. Blue boxes represent the interquartile range, black whiskers extend to the most extreme data points not considered outliers. The median value of each group is show in red. Red plus (+) signs represent outliers. Organs denoted with an asterisk (*) show a statistically significant time decrease under use of AS with a threshold of p<0.025 for the one-tailed Mann–Whitney U test.

AS models resulted in a measurable reduction in median contouring times for several of the OARs. Statistically significant median time reductions – based on a p<0.025 threshold – were observed for the right lung (5.7 min, a 90.7% reduction), left lung (5.9 min, a 92.7% reduction), heart (1.1 min, a 52.9% reduction) and total contouring time (12.6 min, a 50.3% reduction). Median times also decreased, though without reaching statistical significance, for the spinal canal, aorta and right bronchial tree. Conversely, small increases in contouring times were observed for the left bronchial tree, esophagus and trachea. Fig. 2 shows contouring time distributions for all OARs. Additional timing statistics are summarized in Table 1.

### 3.2 Geometric performance metrics

The geometric performance of the AS models is summarized in Table 2. Median DSC values ranged from 0.82 to 0.99, with most OARs achieving scores above 0.85. Median surface DSC exceeded 0.90.

Metrics related to contour surface irregularities, such as totalAPL, were consistently low for the majority of OARs, suggesting that minimal manual adjustments were necessary. Notable exceptions were observed for the aorta and heart, where higher median totalAPL values were recorded.

**Fig. 3:**
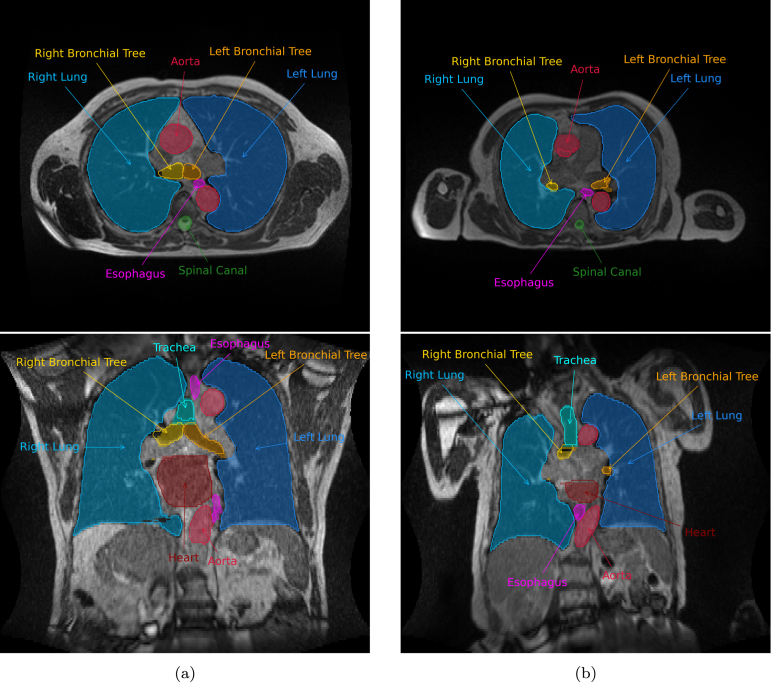
Axial (top) and coronal (bottom) views of the two Group 1 patients with highest and lowest DSC average over all OARs (shown in (a) and (b) respectively) with AS results and corrected contours overlaid. AS results are shown as solid lines, while corrected contours are displayed as shaded areas.

HD, avgHD, and HD95 medians were consistently low for all OARs, with no significant outliers. Fig. 3 shows example AS results and the final corrected OAR contours for 2 exemplary patients.

### 3.3 Correlation between geometric metrics and correction times

The Spearman correlation coefficients and corresponding p-values reveal weak to negligible correlations of individual geometric metrics versus correction times for most organs. Notable exceptions were the aorta, esophagus and left lung, each of which exhibited statistically significant correlations with correction times for four metrics. These results are summarized in Table 3, which provides the correlation coefficients and significance levels for all OARs and metrics.

**Table 1:** Per organ median contouring times for Group 1 and Group 2, difference between the two and p-value for the significance of the potential decrease according to the one-tailed Mann–Whitney U test.

	Group 1 (AS) median times [s]	Group 2 (manual) median times [s]	Difference (Group 1 - Group 2) [s]	% Difference (Group 1 - Group 2) /Group 2	p-value
Aorta	92	116	−24	−20.7%	0.25
Right lung	35	375	−340	−90.7%	<0.01
Left lung	28	384	−356	−92.7%	<0.01
Right bronchial tree	65	85	−20	−23.5%	0.11
Left bronchial tree	86	70	16	＋22.9%	0.81
Heart	56	119	−63	−52.9%	0.018
Spinal canal	36	64	−28	−43.8%	0.05
Esophagus	161	146	15	＋10.3%	0.65
Trachea	87	72	15	＋20.8%	0.88
Total	750	1509	−759	−50.3%	<0.01

**Table 2:** Per organ medians of segmentation quality metrics based on extent of manual correction for Group 1 patients. IQR of distribution shown in parentheses.

	DSC	surfDSC	totalAPL [mm]	HD [mm]	HD95 [mm]	avgHD [mm]
Aorta	0.94 (0.04)	0.97 (0.06)	139.2 (212.9)	11.1 (15.2)	3.1 (3.4)	0.8 (0.7)
Right lung	0.99 (0.00)	1.00 (0.01)	28.4 (147.0)	4.5 (6.7)	1.5 (0.0)	0.5 (0.2)
Left lung	0.99 (0.01)	1.00 (0.03)	35.9 (409.6)	6.0 (9.7)	1.5 (1.5)	0.4 (0.5)
Right bronchial tree	0.83 (0.04)	0.93 (0.03)	28.6 (26.9)	8.2 (4.8)	3.8 (1.5)	1.2 (0.5)
Left bronchial tree	0.82 (0.08)	0.97 (0.06)	6.0 (35.9)	9.2 (4.5)	3.2 (2.8)	0.9 (0.8)
Heart	0.97 (0.01)	0.93 (0.09)	441.0 (771.0)	10.9 (5.8)	5.3 (3.0)	1.4 (0.6)
Spinal canal	0.87 (0.07)	0.95 (0.06)	6.0 (18.0)	12.1 (15.4)	3.9 (4.0)	0.9 (0.6)
Esophagus	0.85 (0.14)	0.91 (0.12)	22.5 (30.1)	18.3 (10.1)	6.5 (6.9)	1.3 (1.1)
Trachea	0.88 (0.02)	0.96 (0.08)	40.4 (87.3)	8.1 (7.5)	4.5 (3.0)	1.2 (0.6)

**Table 3:** Per organ Spearman correlation coefficients of segmentation quality metrics based on extent of manual correction to times consumed for these correction on Group 1 patients. p-values for correlation significance shown in brackets. Cells with p<0.025 are indicated in bold.

	DSC	surfDSC	totalAPL	HD	HD95	avgHD
Aorta	**−0.76 [0.03]**	−0.62 [0.10]	0.64 [0.09]	**0.81 [0.02]**	**0.76 [0.03]**	**0.81 [0.02]**
Right lung	−0.35 [0.36]	−0.42 [0.27]	0.08 [0.83]	0.45 [0.22]	0.29 [0.45]	0.25 [0.52]
Left lung	−0.43 [0.24]	**−0.83 [0.01]**	**0.73 [0.03]**	**0.79 [0.01]**	0.62 [0.07]	**0.72 [0.03]**
Right bronchial tree	0.51 [0.16]	0.10 [0.80]	0.25 [0.51]	0.08 [0.83]	−0.07 [0.86]	−0.19 [0.63]
Left bronchial tree	0.60 [0.09]	0.53 [0.14]	−0.52 [0.15]	−0.52 [0.15]	−0.42 [0.27]	−0.58 [0.10]
Heart	−0.15 [0.70]	−0.18 [0.64]	0.38 [0.31]	−0.27 [0.49]	−0.23 [0.55]	0.10 [0.80]
Spinal canal	−0.58 [0.10]	−0.49 [0.18]	**0.77 [0.02]**	0.57 [0.11]	0.52 [0.15]	0.63 [0.07]
Esophagus	**−0.82 [0.01]**	**−0.78 [0.01]**	0.41 [0.28]	0.60 [0.09]	**0.79 [0.01]**	**0.80 [0.01]**
Trachea	−0.32 [0.41]	−0.05 [0.90]	−0.65 [0.06]	0.28 [0.46]	0.03 [0.95]	0.38 [0.31]

## 4 Discussion

We evaluated the utility of in-house DL-based AS models in the MRgRT workflow for lung cancer patients. We addressed the lack of commercial solutions for 0.35 T MRgRT, and the time consuming manual segmentation procedure for lungs in the clinical TPS, where no automatic option is available. Our findings demonstrate both strengths and limitations of the AS models in terms of time efficiency, geometric performance, and correlations between geometric metrics and correction times.

The AS models significantly reduced contouring times for several OARs, with substantial improvements observed for the left and right lungs (92.7% and 90.7%, respectively) and for total contouring time (50.3%). This agrees with literature where 50% to 60% time were estimated [29], [30].

The findings also revealed non-significant reductions for the spinal canal, aorta, and right bronchial tree, as well as small increases in contouring times for the left bronchial tree, esophagus, and trachea. These results suggest either that the model performs inconsistently across different anatomical regions or that varying levels of scrutiny are applied per anatomical region and OAR. The increases in correction times for certain structures could stem from their complex or hard to image anatomy and the higher precision required for clinical acceptability.

The geometric performance of the AS models was robust for most OARs, with median DSC values exceeding 0.85 and surface DSC values surpassing 0.90 for all structures. HD was consistently low, indicating that the models generally aligned well with clinicians. These findings reaffirm the accuracy of the AS models and agree with their offline evaluations [16]. This held true for the three additional models trained for this study, despite their comparatively lower performance in offline testing, which is likely caused by a combination of lower training and test data availability, as well as potential inconsistencies in contouring style for these organs. In particular, a high variability in the depth to which both bronchial trees are delineated and the location of the transition between those and the trachea were observed. The same effects likely explain the comparatively higher scores for these OARs in the online cases, with physicians following AS suggestions more closely when the OARs were not close to the target.

Exceptions were observed for the aorta and heart, with higher median totalAPL. These anomalies were primarily due to missing slices in the auto-segmentations for these structures, which were present in 5 cases with one to three missing slices each and disproportionately impacted totalAPL. Such errors highlight a potential limitation in the model’s ability to handle large or irregular structures, or a lack of training samples of the specific regions.

Weak to negligible correlation between geometric performance metrics and manual correction times was observed for most OARs, consistent with findings from previous offline timing studies [18], [26]. While strong geometric alignment, as indicated by high DSC values, often implies reduced correction effort, the results suggest that this relationship is not universally reliable. The lack of correlation could arise from

(i) critical region sensitivity: small errors in clinically significant regions, such as close to tumor margins or high-dose adjacent OARs, may disproportionately increase correction times despite high overall geometric accuracy.
(ii) Anatomical complexity: structures like the esophagus and bronchial tree, with complex or irregular shapes, require greater manual intervention even when geometric metrics are acceptable.
(iii) Subjective physician judgment: individual preferences and variability in delineation styles may further decouple geometric accuracy from time savings. This effect would only be possible to investigate in multi-observer studies.

Exceptions to this trend were observed for the aorta, esophagus, and left lung, where significant correlations were found between correction times and multiple geometric metrics. Thus, for some OARs, traditional metrics like DSC and HD can serve as indicators of clinical efficiency. However, the overall findings reinforce the need for alternative evaluation frameworks that integrate clinical relevance and workload considerations, as there is no obvious way to predict beforehand – at the time of model adoption – which organs will or will not exhibit such discrepancies between geometric performance and correction effort.

This study reinforces previously reported limitations of relying solely on geometric metrics to evaluate AS models. These metrics may fail to capture the nuanced relationship between initial segmentation quality and the manual correction effort required. In line with earlier work [26], [31], [32], we advocate for a more comprehensive evaluation approach that includes:

(i) Correction effort metrics: quantifying the extent of manual edits.
(ii) Region-specific analysis: prioritizing high-impact regions that influence clinical decision-making (proximity to tumors or sensitive OARs).
(iii) Physician feedback: incorporating subjective assessments of contour usability to contextualize quantitative metrics.

The integration of AS models into the MRgRT workflow was facilitated by a semi-automated pipeline. Despite these improvements, manual steps (e.g., USB file transfer) remain necessary due to system limitations. These manual operations introduce potential inefficiencies and could be addressed in future iterations of the pipeline through direct integration with the TPS.

The docker-based architecture demonstrated scalability and adaptability, enabling secure deployment in the clinical environment. This modular approach is extendable to other clinical settings and other model types, regardless of their software requirements. However, additional efforts, some requiring a degree of software development on the clinical machines, are needed to fully automate the process and minimize reliance on manual operations.

To enhance the performance and clinical utility of AS models for MRgRT, future work should focus on

(1) expansion from planning to fractions: while time savings in treatment planning are valuable, extending AS to fraction-level MRgRT workflows could deliver the greatest impact. Online AS for fraction scans would reduce treatment times, lower scan-to-irradiation intervals, enhancing efficiency and patient comfort, as has already been shown on systems which support importing of contours for fraction scans [33]. Realizing this is unfortunately dependent on software modifications in the online adaptive workflow of the clinical TPS.
(2) Model refinement and safety training: develop architectures and training protocols tailored to complex anatomies, emphasizing error minimization in critical regions. Models should be designed to handle edge cases, identify areas of uncertainty, and flag results for additional review when necessary, ensuring patient safety.
(3) Comprehensive evaluation frameworks: combining geometric, clinical, and workload metrics to provide an assessment of model performance, ensuring evaluation criteria capture the nuances of clinical practice. Utilities tracking the interaction of clinicians with AI results should be part of commercial solutions. This would enable tracking of the value provided by each system over its lifetime.
(4) Longitudinal impact studies: evaluating the long-term effects of AS-assisted workflows on clinician workload, patient throughput, and treatment outcomes. Such studies should also examine behavioral changes as clinicians grow accustomed to relying on AI-generated contours, potentially shifting levels of trust and scrutiny over time.
(5) Compatibility and open protocols: encouraging the development of clinical machines and treatment planning systems that support seamless integration with AS models through open protocols and standardized communication frameworks. This would facilitate interoperability, reduce manual intervention, and enable broader adoption of AI-assisted workflows across different clinical environments.

## Declaration of competing interest

The authors declare that they have no known competing financial interests or personal relationships that could have appeared to influence the work reported in this paper.

Guillaume Landry is an Editorial Board Member for this journal and was not involved in the editorial review or the decision to publish this article.
